# 

**Published:** 1879-12

**Authors:** 


ESTABLISHED IN 1855.

VOLUME XVII. **DECEMBER, 1879.** NUMBER 9

### ATLANTA MEDICAL AND SURGICAL JOURNAL.

S G O L INDEX

*MONTHLY: THREE DOLLARS A YEAR, IN ADVANCE.*

EDITOR:

J. G. WESTMORELAND, M.D.,

*Professor of Materia Medica in Atlanta Medical College.*

H. H. DICKSON, PUBLISHER.

*PAX ET SCIENTIA, SED VERITAS SINE TIMORE.*

ATLANTA, GEORGIA:

*H. H. DICKSON, BOOK AND JOB PRINTER AND BOOKBINDER.*

1879.

